# Supplementary material for: Species-Specific Paternal Age Effects and Sperm Methylation Levels of Developmentally Important Genes
Source: Cells. 2022 Feb 19;11(4):731. doi: 10.3390/cells11040731 (PMC8870257; doi:10.3390/cells11040731)
Supplement: Supplementary file 1 [file cells-11-00731-s001.zip › cells-1534220-supplementary.pdf]

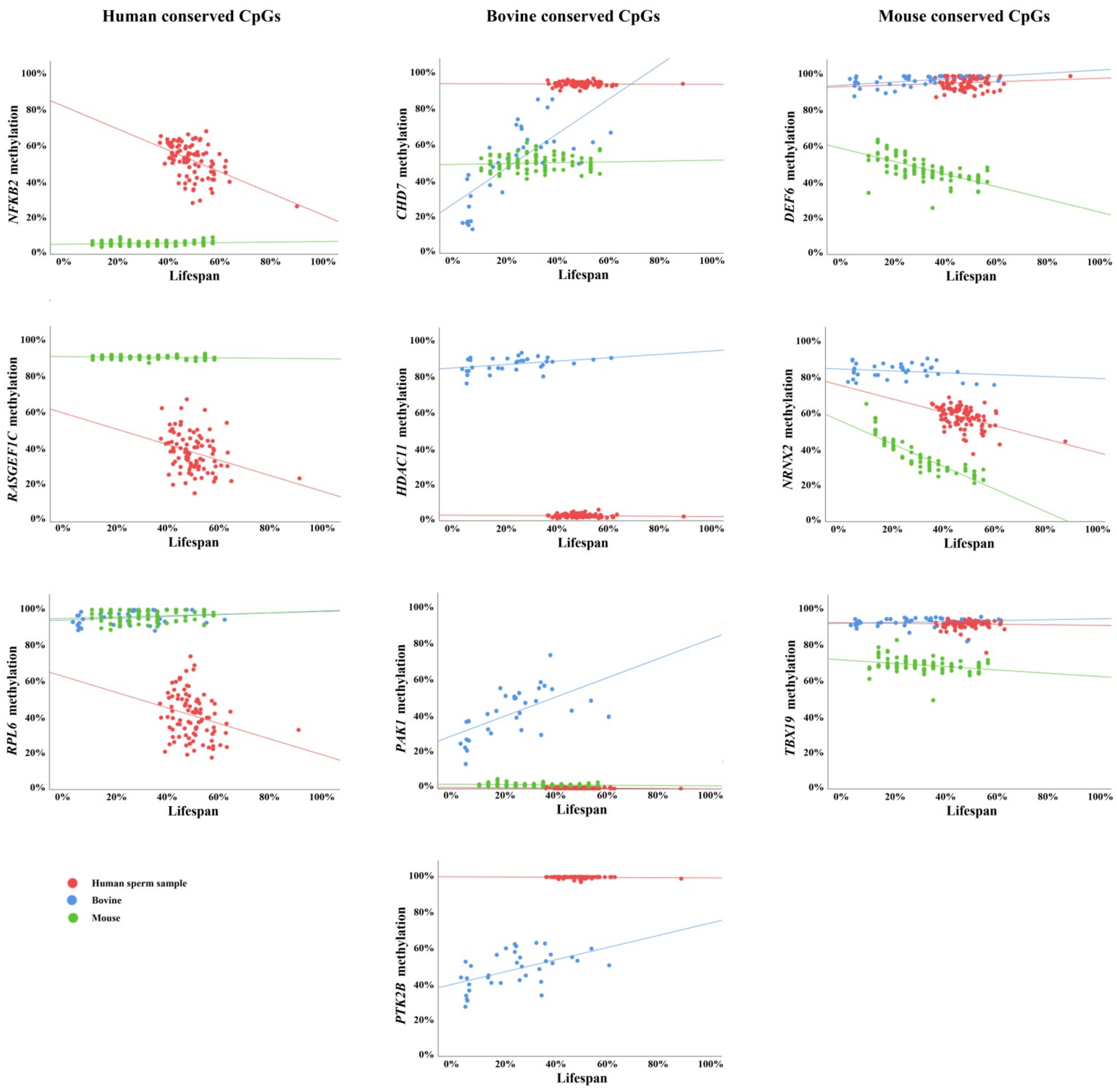

Figure S1. Scatter plots showing the correlations between methylation (y-axis in %) of individual evolutionarily conserved CpGs (see Table below) and donor age (x-axis in percentage of lifespan) in 94 human sperm samples (indicated by red dots), 36 bovine samples (blue dots), and 94 mouse samples (green dots).

**Gene**	**Genomic localization of the analyzed evolutionarily conserved CpG sites**[a]		
	**HSA**	**BTA**	**MMU**
NFKB2	chr10: 102,398,797	n.d.	chr19: 46,308,688
RASGEF1C	chr5: 180,128,418	n.d.	chr11: 49,960,589
RPL6	chr12: 112,408,247	chr17: 61,838,769	chr5: 121,205,847
CHD7	chr8: 60,742,015	chr14: 26,361,261	chr4: 8,752,069
HDAC11	chr3: 13,481,297	chr22: 58,440,704	n.d.
PAK1	chr11: 77,411,819	chr29: 18,586,641	chr7: 97,843,091
PTK2B	chr8: 27,397,612	chr8: 74,491,017	n.d.
DEF6	chr6: 35,309,739	chr23: 9,282,385	chr17: 28,217,034
NRXN2	chr11: 64,636,329	chr29: 42,885,109	chr19: 6,504,094
TBX19	chr1: 168,291,254	chr3: 253,081	chr1: 165,153,631

[a] Genome Reference Consortium Human Build 38 (GRCh38)/hg38, ARS-UCD1.2/bosTau9, and Genome Reference Consortium Mouse Build 38 (GRCm38)/mm10 were used as references.

Table S1. Genes with age-related differentially methylated regions, identified by RRBS in human, bovine, and mouse sperm samples.

Gene	RRBS	Genomic location[a]	Average Methylation	Differential Methylation	Gene region	Gene function
NFKB2	Human ageDMRs	HSA10: 102,398,797–102,398,849	44%	-1.2%	Promoter	Subunit of the transcription factor complex NFkB; central activator of genes involved in inflammation and immune functions.
RASGEF1C		HSA5: 180,128,402–180,128,452	58%	-1.0%	CTCF binding	Guanine-nucleotide releasing factor, involved in small GTPase mediated signal transduction.
RPL6		HSA12: 112,408,247–112,408,263	58%	-1.4%	Promoter	Component of the 60S ribosomal subunit, participating in ribosome assembling; ribosome-independent functions in DNA damage response and immunosurveillance.
CHD7	Bovine ageDMRs	BTA14: 26,361,261–26,361,323	55%	8.5%	Promoter	Transcriptional regulator that contains several helicase family domains; associated with CHARGE syndrome.
HDAC11		BTA22: 58,440,641–58,440,645	93%	2.4%	Promoter	Class IV histone deacetylase; involved in epigenetic repression and transcriptional regulation.
PAK1		BTA29: 18,586,586–18,586,667	52%	6.6%	Promoter	Member of serine/threonine-protein kinase family; involved in intracellular nuclear signaling pathways, cytoskeleton reorganization and cell adhesion.
PTK2B		BTA8: 74,490,924–74,490,972	66%	4.0%	Promoter	Cytoplasmic protein tyrosine kinase; participates in calcium-dependent regulation of ion channels; activates MAP kinase signaling.
DEF6	Mouse ageDMRs	MMU17: 28,217,012–28,217,035	34%	-16.2%	Promoter	Candidate gene for schizophrenia.
NRXN2		MMU19: 6,503,984–6,504,132	58%	-21.5%	Protein coding	Member of the neurexin gene family; functions as a cell adhesion molecule and receptor in the vertebrate nervous system.
TBX19		MMU1: 165,153,599–165,153,641	68%	-3.6%	Promoter flanking	Member of a transcription factor family that share a common DNA-binding domain (T box) and regulate the expression of pituitary POMC.

[a] Genome Reference Consortium Human Build 38 (GRCh38)/hg38, ARS-UCD1.2/bosTau9, and Genome Reference Consortium Mouse Build 38 (GRCm38)/mm10 were used as references.

Table S2. PCR and sequencing primers for bisulfite pyrosequencing in human, bovine, and mouse sperm.

Gene	Primer	Sequence (5'-3')[a]	Location[b]	No. of CpGs	Annealing Temp. (^{o}C)
**Human ageDMRs**					
HSA *NFKB2*	Forward	GGAGGAAGGTTTTGTTTATTTTTTTTTAGT	HSA chr10: 102,398,763 – 102,398,895		60
	Reverse	*TTTACCCCTCCCTCCATCAATAC			
	Sequencing	AGGTTTTGTTTATTTTTTTTTAGTT		3	
MMU *Nfkb2*	Forward	TTTTAGATAAGGAGGAAGTGTAGAG	MMU chr19: 46,308,623 – 46,308,783		60
	Reverse	*CCTTCTCCCTACCACTTACA			
	Sequencing	AGGTTTTGTTTATTTTTTTTTAGTT		4	
HSA *RASGEF1C*	Forward	GGGAGGGTATAGTGTATTTGTGT	HSA chr5: 180,128,340 – 180,128,486		62
	Reverse	*CCTCCATCCACAAAAAACTCCTTAAT			
	Sequencing	GGTGAGGAAGGTGGT		3	
MMU *Rasgef1c*	Forward	GGAGTTTTTGGGTGTTTGTAGTG	MMU chr11: 49,960,535 – 49,960,620		60
	Reverse	*TCTTTCCCTAAATCCCACTCAC			
	Sequencing	GGTGTTTGTAGTGATTTT		2	
HSA *RPL6*	Forward	GTAATTGTTATAAAATTAGTTGGTGGTGA	HSA chr12: 112,408,214 – 112,408,308		60
	Reverse	*TCCAATTTACAATCCCCACATC			
	Sequencing	AGTTGGTGGTGATAAGAA		4	
BTA *RPL6*	Forward	AGTTGGTGGAGATAAGAATGG	BTA chr17: 61,838,741 – 61,838,919		60
	Reverse	*TAAACCCCAAACTCACTATCCT			
	Sequencing	AGATAAGAATGGTGGTAT		2	

MMU *Rpl6*	Forward	ATTAAAATAGTTGGTGGGATAAGA	MMU chr5:121,205,796 – 121,205,886		60
	Reverse	*CCCAAAAAAAACAACCCAAACT			
	Sequencing	GTTGGTGGGATAAGAA		3	
**Bovine ageDMRs**					
BTA *CHD7*	Forward	GGGTAGGGTTATTTTTTATATTTGT	BTA chr14: 26,361,213 – 26,361,349		58
	Reverse	*TTTCCCTAACTCAAACCCTCTTA			
	Sequencing 1	GGTTATTTTTTATATTTGTAGTAGT		3	
	Sequencing 2	GTAGTATGGTTAGTTATAGT		2	
HSA *CHD7*	Forward	GGTTTATTTTTTGTTGTGGTTGATTAT	HSA chr8: 60,741,869 – 60,742,066		60
	Reverse	*ACCCCAAAACTATTCAAATACCA			
	Sequencing 1	ATGTTGTTGTATGGAAAAATT		1	
	Sequencing 2	GGTTTTGTGTAGGGG		5	
MMU *Chd7*	Forward	ATGTAGTAGATGGGTAGTTATTTGG	MMU chr4: 8,752,042 – 8,752,156		60
	Reverse	*TTCCCTAACTAAAACCCTCTTAACCTTAAA			
	Sequencing	AGATGGGTAGTTATTTGGTA		2	
BTA *HDAC11*	Forward	TGGGTTGTAGGGAGTAGAT	BTA chr22: 58,440,562 – 58,440,749		58
	Reverse	*CCAACCCAACTATACCAACA			
	Sequencing 1	GTTGTAGGGAGTAGATGTA		2	
	Sequencing 2	GAGGTTGATTATTTTGTTTTATT		2	
	Sequencing 3	GGTTTAGGAAGGTTATGTT		7	
HSA *HDAC11*	Forward	GGGGGATTTTTTATATTTTTTAGGAAATT	HSA chr3: 13,481,257 – 13,481,410		58
	Reverse	*CCCAACTATACCAACATATACCAAAAACA			
	Sequencing 1	AAATTGATTATTTTGTTTTATTT		1	
	Serquencing 2	GTTTATGAAGGTGATGTTGTA		5	

BTA *PAK1*	Forward	ATGTTTTAGGGTGGGTTAGTATTAT	BTA chr29: 18,586,469 – 18,586,712		60
	Reverse	*CAACCCCAAAACTAAACCTTTACT			
	Sequencing 1	GGGTGGGTTAGTATTATT		6	
	Sequencing 2	GATATTTAGGTTTTATT		5	
HSA *PAK1*	Forward	GGTATTATTTGGTGGGGAAGGTTAG	HSA chr11: 77,411,766 – 77,411,908		62
	Reverse	*CCAAAAACCCAACCCAACTAAATC			
	Sequencing 1	GGGGAAGGTTAGTTT		5	
	Sequencing 2	GGGTATTTATAAGGTTTTTGTTTT		3	
MMU *Pak1*	Forward	TTTTAATTGTTGAGTAGGGAGAGG	MMU chr7: 97,843,057 – 97,843,195		60
	Reverse	*TTCTACCCCAACTCTCAATCTAACTATA			
	Sequencing	TGAGTAGGGAGAGGT		2	
BTA *PTK2B*	Forward	GGGTTTTGGGGGGTTTTTA	BTA chr8: 74,490,878 – 74,491,040		60
	Reverse	*AAATAAACTCACACCCATCATTTC			
	Sequencing 1	GGGGGGTTTTTAGGT		3	
	Sequencing 2	GGTTTAGGGTTTTAGATA		3	
	Sequencing 3	GGTTAGGATTAGTATAT		2	
HSA *PTK2B*	Forward	TTTGGGGTTATGAGGTATGTG	HSA chr8: 27,397,480 – 27,397,678		60
	Reverse	*CTACTAATACCACCACCATAAACTCTA			
	Sequencing 1	GTTGTTTTTGTAGGATTGTAAT		1	
	Sequencing 2	GGATGTTTGGGGTGT		5	
**Mouse ageDMRs**					
MMU *Def6*	Forward	AGTTTTTTGGTTAAGGTTTGATT	MMU chr17: 28,216,907 – 28,217,114		58
	Reverse	*ACTCCTCACCTTATCTAAAATATACTT			
	Sequencing	ATATTGTTTTGAATATTTTTTATG		4	

HSA *DEF6*	Forward	GGTAGGGTATGTATTTTTGGTTGGATAT	HSA chr6: 35,309,647 – 35,309,787		62
	Reverse	*ACTACCACTCTACTCTATCCCAAATACT			
	Sequencing	TTGGTTGGATATAGGG		4	
BTA *DEF6*	Forward	ATGTATTTGTTGAGGTAGGGTATGT	BTA chr23: 9,282,311 – 9,282,446		60
	Reverse	*CTCAAATACTATCCCACAACCT			
	Sequencing	GGTATGTATTTTTGGTTGG		6	
MMU *Nrxn2* assay1	Forward	*GGGGTTTATTTTGGGGTTGAG	MMU chr19: 6,504,071 – 6,504,151		58
	Reverse	CCCACCCTCTCTCCTCTT			
	Sequencing	CCCTCTCTCCTCTTC		4	
MMU *Nrxn2* assay 2	Forward	ATAGAGAGGGATGTAGGTAAGG	MMU chr19: 6,503,948 – 6,504,228		58
	Reverse	*TCTAATCCTTCAAAACTCCCAACAAAAC			
	Sequencing	GGGTGTTAGGTTTTTA		4	
HSA *NRXN2*	Forward	TGGGGTTTATTTTGGGGTTGAG	HSA chr11: 64,636,266 – 64,636,354		60
	Reverse	*TCTCTCCCCTTCTCCTTCTCTT			
	Sequencing	ATTTTGGGGTTGAGAT		4	
BTA *NRXN2*	Forward	GGGGTTTATTTTGGGGTTGAG	BTA chr29: 42,885,053 – 42,885,133		58
	Reverse	*CCCCTCTATCTCCTCTTTACCAT			
	Sequencing	ATTTTGGGGTTGAGAT		4	
MMU *Tbx19*	Forward	*TGAATGTAGATATAGTTGTGGTTAGAGAT	MMU chr1: 165,153,556 – 165,153,692		60
	Reverse	AAACTAAACCCCAATACTATATACTCTCT			
	Sequencing	ACTCTCTCCTACTAAACT		6	
HSA *TBX19*	Forward	*ATGGGAGTTTTTATTTAGTGGGTTTTA	HSA chr1: 168,291,215 – 168,291,375		58
	Reverse	TACTCCCTCCTACTAAACTTTATCC			
	Sequencing	CCTACTAAACTTTATCCCT		5	

BTA *TBX19*	Forward	*TTGGGTGGATGTAGATATAGTTG	BTA chr3: 253,001 – 253,132		60
	Reverse	CCAATACCATATACTCACTCCTTCTAA			
	Sequencing	CCTTCTAAACTTTATCCCA		3	

[a] Primers indicated by a star are biotinylated at the 5' end.

[b] Genome Reference Consortium Human Build 38 (GRCh38)/hg38, ARS-UCD1.2/bosTau9, and Genome Reference Consortium Mouse Build 38 (GRCm38)/mm10 were used as references.

Table S3. Correlation between single CpG methylation and donor age in human, bovine, and mouse sperm.

CpGs which are conserved between species are highlighted in different colours.

Human ageDMRs

	Human			Mouse			
NFKB2 assay	1			1			
CpG	1	2	3	1	2	3	4
Genomic location	10:102398797	10:102398839	10:102398848	19:46308688	19:46308693	19:46308726	19:46308744
Correlation coefficient	-0.48	-0.25	-0.40	0.14	0.19	0.15	0.15
p value	0.00	0.07	0.01	0.20	0.07	0.16	0.15

	Human			Mouse	
RASGEF1C assay	1			1	
CpG	1	2	3	1	2
Genomic location	5:180128402	5:180128418	5:180128451	11:49960567	11:49960589
Correlation coefficient	-0.31	-0.32	-0.30	-0.04	-0.19
p value	0.01	0.01	0.04	0.74	0.06

	Human				Bovine		Mouse		
RPL6 assay	1				1		1		
CpG	1	2	3	4	1	2	1	2	3
Genomic location	12:112408272	12:112408269	12:112408262	12:112408247	17:61838784	17:61838769	5:121205822	5:121205832	5:121205847
Correlation coefficient	-0.25	-0.25	-0.24	-0.26	0.22	0.30	-0.01	-0.09	0.14
p value	0.05	0.05	0.06	0.04	0.19	0.08	0.92	0.39	0.19

Bovine ageDMRs

	Bovine							
CHD7 assay	1			2				
CpG	1	2	3	1	2			
Genomic location	14:26361248	14:26361261	14:26361280	14:26361301	14:26361322			
Correlation coefficient	0.74	0.75	0.76	0.73	0.79			
p value	0.00	0.00	0.00	0.00	0.00			
	Human						Mouse	
	1	2					1	
	1	1	2	3	4	5	1	2
	8:60742015	8:60741956	8:60741953	8:60741947	8:60741941	8:60741932	4:8752069	4:8752095
	-0.01	0.23	-0.10	0.05	-0.09	-0.04	0.08	0.04
	0.94	0.07	0.43	0.69	0.48	0.73	0.42	0.74

	Bovine										
HDAC11 assay	1		2		3						
CpG	1	2	1	2	1	2	3	4	5	6	7
Genomic location	22:58440588	22:58440593	22:58440641	22:58440644	22:58440691	22:58440693	22:58440696	22:58440704	22:58440715	22:58440717	22:58440723
Correlation coefficient	0.12	0.19	0.25	0.52	0.27	0.17	0.27	0.37	0.36	0.25	0.30
p value	0.49	0.26	0.14	0.00	0.13	0.34	0.13	0.03	0.04	0.16	0.08
	Human										
	1	2									
	1	1	2	3	4	5					
	3:13481360	3:13481310	3:13481308	3:13481305	3:13481297	3:13481286					
	-0.19	-0.10	-0.24	-0.31	-0.05	-0.11					
	0.13	0.41	0.05	0.01	0.69	0.41					

	**Bovine**										
PAK1 assay	1						2				
CpG	1	2	3	4	5	6	1	2	3	4	5
Genomic location	29:18586681	29:18586672	29:18586666	29:18586651	29:18586641	29:18586633	29:18586586	29:18586562	29:18586556	29:18586542	29:18586538
Correlation coefficient	0.59	0.57	0.55	0.67	0.64	0.57	0.60	0.60	0.61	0.65	0.68
p value	0.00	0.00	0.00	0.00	0.00	0.00	0.00	0.00	0.00	0.00	0.00
	**Human**								**Mouse**		
	1					2			1		
	1	2	3	4	5	1	2	3	1	2	
	11:77411794	11:77411806	11:77411816	11:77411819	11:77411827	11:77411847	11:77411880	11:77411884	7:97843083	7:97843091	
	-0.04	-0.07	-0.04	-0.17	-0.16	0.13	0.04	0.06	0.01	-0.09	
	0.76	0.59	0.78	0.17	0.20	0.30	0.76	0.65	0.94	0.37	

	**Bovine**							
PTK2B assay	1			2			3	
CpG	1	2	3	1	2	3	1	2
Genomic location	8:74491017	8:74491014	8:74490993	8:74490971	8:74490955	8:74490952	8:74490934	8:74490924
Correlation coefficient	0.52	.370*	0.25	0.33	0.32	0.38	0.57	0.56
p value	0.00	0.03	0.15	0.05	0.06	0.02	0.00	0.00
	**Human**							
	1	2						
	1	1	2	3	4	5		
	8:27397561	8:27397599	8:27397612	8:27397628	8:27397633	8:27397636		
	-0.05	0.08	-0.09	-0.08	-0.11	-0.07		
	0.71	0.56	0.50	0.62	0.47	0.66		

Mouse ageDMRs

	Mouse				Human			
DEF6 assay	1				1			
CpG	1	2	3	4	1	2	3	4
Genomic location	17:28217012	17:28217034	17:28217042	17:28217053	6:35309753	6:35309739	6:35309717	6:35309692
Correlation coefficient	-0.69	-0.73	-0.73	-0.55	-0.04	0.11	0.21	-0.15
p value	0.00	0.00	0.00	0.00	0.77	0.38	0.10	0.51
	Bovine							
	1							
	1	2	3	4	5	6		
	23:9282405	23:9282396	23:9282390	23:9282385	23:9282363	23:9282339		
	0.01		0.29	0.49	-0.01	0.19		
	0.96		0.09	0.00	0.95	0.33		

	Mouse							
NRXN2 assay	1				2			
CpG	1	2	3	4	1	2	3	4
Genomic location	19:6504132	19:6504125	19:6504118	19:6504112	19:6504094	19:6504063	19:6504046	19:6503984
Correlation coefficient	-0.25	-0.46	-0.74	-0.58	-0.94	-0.82	-0.52	-0.82
p value	0.02	0.00	0.00	0.00	0.00	0.00	0.00	0.00
	Human				Bovine			
	1				1			
	1	2	3	4	1	2	3	4
	11:64636329	11:64636311	11:64636298	11:64636291	29:42885109	29:42885090	29:42885084	29:42885077
	-0.47	0.03	-0.07	-0.12	-0.12	-0.14	0.06	-0.05
	0.00	0.81	0.58	0.37	0.50	0.42	0.74	0.78

	Mouse							
TBX19 assay	1							
CpG	1	2	3	4	5	6		
Genomic location	1:165153650	1:165153646	1:165153641	1:165153631	1:165153620	1:165153599		
Correlation coefficient	-0.30	-0.35	-0.31	-0.29	-0.35	-0.28		
p value	0.00	0.00	0.00	0.01	0.00	0.01		
	Human					Bovine		
	1					1		
	1	2	3	4	5	1	2	3
	1:168291243	1:168291254	1:168291265	1:168291271	1:168291286	3:253092	3:253081	3:253070
	0.04	-0.03	-0.09	0.08	-0.20	0.39	0.45	0.24
	0.75	0.82	0.49	0.53	0.12	0.02	0.01	0.16
